# BTK Inhibitors and CAR T-Cell Therapy in Treating Mantle Cell Lymphoma—Finding a Dancing Partner

**DOI:** 10.1007/s11912-022-01286-0

**Published:** 2022-05-21

**Authors:** Javier L. Munoz, Yucai Wang, Preetesh Jain, Michael Wang

**Affiliations:** 1 Mayo Clinic, Phoenix, AZ USA; 2 Mayo Clinic, Rochester, MN USA; 3 Department of Lymphoma-Myeloma, Division of Cancer Medicine, MD Anderson Cancer Center, University of Texas, Houston, TX USA

**Keywords:** Bruton’s tyrosine kinase, Chimeric antigen receptor T-cell therapy, Relapsed/refractory mantle cell lymphoma, Combination therapy

## Abstract

**Purpose of Review:**

This review focuses on the feasibility of combining Bruton’s tyrosine kinase (BTK) inhibitors (BTKis) with chimeric antigen receptor (CAR) T-cell therapy in patients with relapsed or refractory (R/R) mantle cell lymphoma (MCL). Potential scenarios for combination treatment with these agents are presented.

**Recent Findings:**

BTKis and CAR T-cell therapy have revolutionized the treatment paradigm for R/R MCL. Ibrutinib, acalabrutinib, and zanubrutinib are covalent irreversible BTKis approved for R/R MCL. Brexucabtagene autoleucel was the first CAR T-cell therapy approved for R/R MCL based on findings from the ZUMA-2 trial. There is evidence to suggest that combination treatment with BTKis and CAR T-cell therapy may improve CAR T-cell efficacy.

**Summary:**

As BTKis and CAR T-cell therapy become mainstays in R/R MCL therapy, combination treatment strategies should be evaluated for their potential benefit in R/R MCL.

## Introduction

The pathogenesis of mantle cell lymphoma (MCL) is driven by various mechanisms, including anomalous cell cycle regulation, dysregulation of B-cell receptor (BCR) signaling, molecular and genomic changes, DNA damage, and microenvironmental effects [1]. As Bruton’s tyrosine kinase (BTK) is a key intermediary in BCR signaling, BTK inhibitors (BTKis) are highly effective in the treatment of MCL [2]. Ibrutinib is a first-in-class BTKi approved for relapsed or refractory (R/R) MCL [2] based on outcomes reported in phase 2 (overall response rate [ORR], 67%; complete response [CR], 23%) and phase 3 trials (ibrutinib vs. temsirolimus, ORR 77 vs. 47%; CR 23 vs. 3%) [3, 4]. Ibrutinib also inhibits interleukin-2 (IL-2) inducible T-cell kinase (ITK), tyrosine protein kinase (TEC), epidermal growth factor receptor (EGFR), Janus kinase 3, and human EGFR 2 [5]. Inhibition of ITK by ibrutinib suppresses the Th 2 response [6] and induces a Th1-dominant response, which promotes cytotoxicity and influences immune surveillance by effector cells that is driven by interferon gamma (IFN-γ) and IL-2 [7]. The second-generation irreversible covalent BTKi acalabrutinib and irreversible covalent BTKi zanubrutinib have minimal inhibition of ITK, TEC, and EGFR [8, 9], resulting in fewer off-target effects than ibrutinib. These agents were efficacious (acalabrutinib, ORR=81%; CR=40%; zanubrutinib, ORR=84%; CR=69%) and safe in patients with R/R MCL [10, 11] and were subsequently approved for this indication.

Following the advent of BTKis, the emergence of CD19-targeted chimeric antigen receptor (CAR) T-cell therapy represents another major advance in R/R MCL therapy. Brexucabtagene autoleucel (brexu-cel; formerly known as KTE-X19) was the first approved CD19-targeted CAR T-cell therapy for R/R MCL based on findings from the phase 2 ZUMA-2 trial, which demonstrated an ORR of 93% and CR rate of 67% [12••]. However, immune-mediated adverse events (AEs) such as cytokine release syndrome (CRS) and neurotoxicity are frequently observed with CAR T-cell therapy [12••]. In ZUMA-2, elevated cytokine levels (i.e., interleukin-6 [IL-6], IL-2, and IFN-γ) correlated with increased severity of CRS and neurotoxic AEs [12••].

A subset of patients may not derive an adequate benefit from BTKi or CAR T-cell therapy when administered alone. Patients may experience treatment intolerance, frequently seen with ibrutinib, leading to discontinuation [13]. Additionally, BTKi resistance stemming from complex genetic and non-genetic mechanisms may develop [14]. The benefit of CAR T-cell therapy may be short-lived in some patients due to insufficient CAR T-cell expansion, T-cell exhaustion, T-cell senescence, resistance due to the preexistence of CD19− clones, and inhibition of T-cell activity [15]. Administration of CAR T-cell therapy after a BTKi was demonstrated in the ZUMA-2 and real-world studies of brexu-cel, where a BTKi was frequently used in bridging therapy regimens in patients previously exposed to a BTKi. Evidence suggests that concomitant administration of BTKi and CAR T-cell therapy may provide a greater treatment benefit than either agent alone [16–21]. In vitro analyses demonstrate that stimulation of CAR T-cells with a BTKi enhances the Th1 response and T-cell effector activity by increasing cytokine production and cytolytic activity [21]. In addition, exposure to a BTKi increases T-cell expansion, viability, and engraftment [16, 17]. Preliminary evidence in patients with R/R chronic lymphocytic leukemia (CLL) further corroborates preclinical observations. Here, we present an overview of BTKi and CAR T-cell therapy in R/R MCL and describe strategies for incorporating BTKi therapy in the CAR T-cell setting, which may help guide clinical decisions and treatment selection.

## BTK Inhibitors for R/R MCL

The BTKis approved for R/R MCL (ibrutinib, acalabrutinib, and zanubrutinib) covalently and irreversibly bind to cysteine 481 within the ATP binding domain [22]. Ibrutinib was approved for R/R MCL following results from an international phase 2 study, which reported an ORR of 67% with 23% CR; median duration of response (DoR) was 17.5 months [3]. The efficacy of ibrutinib in R/R MCL was further confirmed in the phase 3 MCL3001 trial, which [23] demonstrated longer progression-free survival (PFS) with ibrutinib compared with the mTOR inhibitor temsirolimus (hazard ratio [HR] 0.43 [95% CI 0.32–0.58] *P*<0.0001) [23].

To reduce toxicities related to off-target effects of ibrutinib, more selective BTKis were developed. Findings from the phase 2 ACE-LY-2004 study of acalabrutinib in patients with R/R MCL reported an ORR of 81% with 48% CR; at a median follow-up of 38.1 months, median PFS was 22.0 months and median overall survival (OS) was not reached [24]. In a pivotal phase 2 study of zanubrutinib in patients with R/R MCL, the ORR was 84% with 69% CR [11]. At a median follow-up of 3 years, median PFS was 33 months [11]. Among BTKis under development, pirtobrutinib, a highly selective, non-covalent, reversible BTKi designed to override BTKi resistance stemming from C481S mutations, was investigated in a phase 1/2 study in patients with R/R MCL [25]. At a median follow-up of 8.2 months, the ORR in patients previously exposed to a BTKi was 51%, with 25% achieving CR; among BTKi-naïve patients, the ORR was 82% with 18% achieving CR [26].

It should be noted that the ability of a BTKi to penetrate the blood–brain barrier renders it potentially effective in the treatment of patients with relapsed disease with central nervous system (CNS) involvement based on evidence showing that ibrutinib and zanubrutinib improved outcomes in these high-risk patients [27–29].

### Toxicities Associated with BTKi Therapy

Toxicities associated with off-target effects of BTKis frequently lead to treatment discontinuation [30]. Reported rates of discontinuation due to treatment-emergent AEs in clinical trials of ibrutinib range from 7 to 28% [31]; corresponding discontinuation rates are comparably lower in patients receiving acalabrutinib (7–11%) or zanubrutinib (9–13%) [11, 31, 32]. Common AEs associated with BTKi therapy include bruising/bleeding, cardiovascular events, skin rash, and diarrhea [30, 33]. Bleeding events are associated with ibrutinib, with up to 5% being cases of major hemorrhage [30], and may be attributed to dysfunctional glycoprotein VI signaling and repressed collagen-mediated platelet aggregation, likely stemming from inhibition of BTK and other TEC family kinases [5, 34]. Patients receiving ibrutinib have an increased risk for atrial fibrillation, heart failure, ventricular arrhythmias, and supraventricular arrhythmias [35, 36]. In patients receiving ibrutinib, inhibition of off-target kinases and PI3K/AKT signaling pathways essential for the maintenance of cardiomyocytes may contribute to the development of heart failure [36]. Evidence suggests that atrial fibrillation may be due to ibrutinib-induced cardiac fibrosis, atrial enlargement, and dysregulation of calcium flux and repolarization [35]. Diarrhea and dermatologic AEs are observed with ibrutinib and acalabrutinib [37, 38] and may stem from an inhibitory effect on EGFR [5, 38]. An increased risk of infection in patients receiving ibrutinib may stem from its dual inhibitory effect on BTK in macrophages and ITK in T-cells [5]. Although neutropenia is more frequently observed with zanubrutinib than with ibrutinib, the rate of infections is not markedly different with either agent [39]. Aspergillosis has also been observed in patients receiving ibrutinib and may stem from the deterioration of fungal immune surveillance following BTK inhibition [40, 41].

### Resistance to BTKis in MCL

Mechanisms of resistance to BTKi therapy vary across B-cell lymphomas. Resistance to BTKis in patients with CLL generally develops due to secondary mutations and chromosomal aberrations [14, 42, 43], as well as non-genetic compensatory mechanisms [14]. The C481S mutation in BTK hinders the binding of covalent BTKis to BTK; gain-of-function mutations in PLCγ2 allow PLCγ2 to be activated in the absence of BTK [14]. In MCL, the gain of chromosome arm 17q has been observed in patients refractory to ibrutinib [44]. *BIRC5*, which encodes survivin, is located on the 17q arm and is upregulated in resistant lymphoma cells and contributes to their proliferation [44]. *SMARCE1* and *HN1* are also located on the 17q arm and have been implicated in lymphoma cell dissemination [44]. *CARD11* encodes an adaptor protein downstream of the BCR [45]. L878F mutations in *CARD11* have been detected in patients with MCL and other B-cell lymphomas [45]. Mutant *CARD11* expression induces constitutive activation of NF kappa B, which is essential for B-cell survival [45]. Activation of the NF kappa B pathway independent of BCR signaling also plays a role in the development of resistance to BTKi therapy in MCL [46]. Specifically, CD40L is a ligand that binds to CD40, a member of the tumor necrosis factor receptor family, and is an important mediator of B-cell proliferation and differentiation and development of lymphoma [46]. As such, CD40L induces activation of the NF kappa B pathway in MCL cell lines in a non-BCR–dependent manner [46].

#### CAR T-Cell Therapy for MCL

T-cell therapy entails the infusion of genetically engineered autologous T-cells expressing receptors against tumor-cell surface antigens [47]. The CAR typically consists of an extracellular domain enabling tumor antigen recognition connected to individual or multiple intracellular co-stimulatory domains; together, these components induce T-cell activation [47]. Existing CAR T-cell therapies for B-cell non-Hodgkin lymphoma (B-NHL) are directed toward the CD19 surface antigen on B lymphocytes [48]. Because the time from leukapheresis to CAR T-cell infusion can take several weeks [49], patients awaiting CAR T-cell infusion are at risk for disease progression, which sometimes requires bridging therapy to stabilize the disease during the interim period. The selection of appropriate bridging therapy varies based on a patient’s disease characteristics, disease stage, performance status, response to prior therapy, and comorbidities [49]. Bridging therapy was administered in 37% of patients in ZUMA-2 [12••] and in 67% of patients in a real-world study of brexu-cel in R/R MCL [50••], with BTKi-based regimens being the most common in both studies (Table 1). Lymphodepletion chemotherapy is administered to patients a few days prior to CAR T-cell infusion to enhance the efficacy of CAR T-cell therapy and varies by CAR T-cell product [49].

**Table 1:** Clinical studies of CAR T-cell therapy incorporating BTK inhibitors

**Studies in MCL**						
**Reference/study ID**	**Study name/design**	**Patient population**	**CAR T-cell therapy**	**Prior treatment** characteristics	**Bridging therapies**	**Response and safety outcomes**
Wang M, et al [12••, 51] NCT02601313	ZUMA-2 Prospective Phase 2	R/R MCL *N*=68	Brexu-cel	• BTKi, anti-CD20 mAb, or chemotherapy • Refractory to BTKi: 62% • Relapsed on BTKi: 26%	• Any bridging therapy: 37% o Ibrutinib: 21% o Dexamethasone: 18% o Acalabrutinib: 7% o Methylprednisolone: 3% o Ibrutinib + corticosteroid: 6% o Acalabrutinib + corticosteroid: 3%	Response outcomes • ORR: 93% • CR: 67% • ORR by bridging therapy use o With bridging therapy: 90% o Without bridging therapy: 95% • Median TTR: 1 month • Median TTCR: 3 months • Median DoR, PFS, and OS not reached (median FU: 17.5 months) • 12-month PFS: 61% • 12-month OS: 83% Safety outcomes • Cytopenia: 94% • Infections: 32% • CRS: o Any grade: 91% o Grade ≥3: 15% • Neurotoxic AEs: o Any grade: 63% o Grade ≥3: 31%
Palomba ML, et al [52]^a^ NCT02631044	TRANSCEND NHL 001 Prospective Phase 1	R/R MCL subgroup *N*=32	Liso-cel	• BTKi, anti-CD20 mAb, or chemotherapy • BTKi: 88% • Refractory to BTKi: 34%	• Any bridging therapy: 53%	Response outcomes • ORR: 84% • CR: 59% • Median TTCR: 1 month Safety outcomes • Grade ≥3 cytopenias: o Neutropenia: 41% o Anemia: 34% o Thrombocytopenia: 31% • CRS: o Any grade: 50% o Grade ≥3: 3% • Neurotoxic AEs: o Any grade: 28% o Grade ≥3: 9%
Wang Y, Jain P, et al [53]	US Lymphoma CAR-T Consortium	R/R MCL *N*=95	Brexu-cel	• BTKi: 82% • Venetoclax: 35% • Lenalidomide: 23%	• Any bridging therapy: 67% o BTKi-based: 31% o R-chemotherapy ± corticosteroid: 25% o BTKi + venetoclax-based: 14% o Venetoclax ± CD20 antibody: 8% o Radiation ± corticosteroid: 6% o R-chemotherapy + radiation: 5% o Lenalidomide-based; 5% • Corticosteroid: 5% • Corticosteroid + CD20 antibody: 2%	Response outcomes • ORR: 89% • CR: 81% • ORR by bridging therapy use o With bridging therapy: 91% o Without bridging therapy: 87% • CR by bridging therapy use o With bridging therapy: 83% o Without bridging therapy: 77% • Median TTBR: 30 days • Median DoR, PFS, and OS not reached (median FU: 6.7 months) Safety outcomes • CRS: o Any grade: 91% o Grade ≥3: 8% • Neurotoxic AEs: o Any grade: 60% o Grade ≥3: 35%
Romancik JT, et al [54, 55]	Multicenter US Retrospective	Relapsed MCL *N*=66	Brexu-cel	• BTKi: 98%	• BTKi • BTKi + venetoclax • Chemotherapy • Venetoclax • Radiation only • Corticosteroid only • Lenalidomide + R	Response outcomes • ORR: 86% • CR: 77% • Median FU: 4.1 months • 6-month PFS rate: 77% • 6-month OS rate: 88% Safety outcomes • CRS o Any grade: 86% o Grade ≥3:12% • Neurotoxic AEs o Any grade: 61% o Grade ≥3: 35% • Grade ≥3 neutropenia: 37% • Grade ≥3 thrombocytopenia: 43% • Infection: 18%
Herbaux C, et al [56]	French DESCAR-T Registry and LYSA Group	R/R MCL *N*=47	Brexu-cel	• All received prior BTKi and chemoimmunotherapy	• Any bridging therapy: 87%	Response outcomes • ORR: 88% • CR: 62% • Median PFS: 5.3 months • 6-month PFS rate: 58% Safety outcomes • CRS: 79% • Neurotoxic AEs: 49% • Grade ≥3 CRS or neurotoxic AE: 9%
Liu M, et al [18]	Pilot	Refractory MCL (*n*=3) Refractory FL/tFL (*n*=4)	Ibrutinib + CAR T-cell therapy	• All patients had received prior CAR T-cell therapy	• All patients received ibrutinib	Response outcomes • Six patients achieved CR o R/R MCL, *n*=3 o R/R FL, *n*=3 Safety outcomes • CRS events were grade 2-4 • One patient had a grade 2 neurotoxic AE
**Studies in CLL**						
**Reference/study ID**	**Study type**	**Patients/assays/models**	**Treatment**	**Results**		
Turtle CJ, et al [57] NCT01865617	Phase 1/2	R/R CLL after prior ibrutinib *N*=24	CAR T-cell therapy after ibrutinib	• ORR across all patients: 71% o Restaged patients after one CAR T-cell infusion (*n*=19): ORR=74%; CR=21% ▪ Ibrutinib-refractory subgroup (*n*=16): ORR=69%; CR=25% o Patients who received two CAR T-cell infusions (*n*=6): CR=33% (*n*=2/6)		
Gauthier J, et al [19] NCT01865617	Subgroup Analysis	R/R CLL *N*=30	CAR T-cell therapy + ibrutinib^a^ vs. CAR T-cell therapy alone	• Concomitant treatment with ibrutinib and CAR T-cell therapy resulted in an ORR of 83%; patients who did not receive ibrutinib had ORR=56% • CAR T-cell expansion was significantly greater in responders and patients achieving MRD negativity • Concomitant administration of ibrutinib and CAR T-cell therapy was associated with less severe CRS and lower cytokine levels than administration of CAR T-cell therapy alone		
Siddiqi T, et al [58] NCT03331198	TRANSCEND CLL 004 Phase 1	R/R CLL/SLL *N*=23	Liso-cel after BTKi	• ORR=82% (dose level 1: ORR=78%; dose level 2: ORR=85%) • CR/CRi=45% (dose level 1: CR/CRi=56%; dose level 2: CR/CRi=38%) • CRS any grade: 74%; grade ≥3 CRS: 9% • Neurotoxicity any grade: 39%; grade ≥3 neurotoxicity: 22% • Most common grade ≥3 cytopenias: anemia (74%), thrombocytopenia (70%), neutropenia/decreased neutrophil count (70%), leukopenia (43%)		

^a^Treatment with ibrutinib was initiated ≥2 weeks before leukapheresis and continued up to ≥3 months after CAR T-cell infusion

*AE*, adverse event; *BTKi*, Bruton’s tyrosine kinase inhibitor; *CAR*, chimeric antigen receptor; *CLL*, chronic lymphocytic leukemia; *CR*, complete response; *CRi*, complete response with incomplete hematologic recovery; *CRS*, cytokine release syndrome; *DLBCL*, diffuse large B-cell lymphoma; *DoR*, duration of response; *FL*, follicular lymphoma; *FU*, follow-up; *IFN-γ*, interferon gamma; *IL-2*, interleukin 2; *mAb*, monoclonal antibody; *MCL*, mantle cell lymphoma; *MRD*, minimal residual disease; *ORR*, overall response rate; *OS*, overall survival; *PFS*, progression-free survival; *R*, rituximab; *R/R*, relapsed or refractory; *SLL*, small lymphocytic lymphoma; *tFL*, transformed follicular lymphoma; *TTBR*, time to best response; *TTCR*, time to complete response; *TTR*, time to response

In patients with R/R MCL previously treated with a BTKi, CAR T-cell therapy with brexu-cel or lisocabtagene maraleucel (liso-cel) resulted in high response rates and improved survival [12••, 52]. In ZUMA-2, brexu-cel in patients with R/R MCL resulted in an ORR of 93% with 67% of patients achieving CR (median follow-up, 17.5 months); median PFS, OS, and DoR were not reached [12••, 51, 59] (Table 1) and estimated 12-month PFS and OS rates were 61% and 83%, respectively [12••]. Notably, CAR T-cell expansion was substantially greater after prior ibrutinib than after prior acalabrutinib alone [60•]. In ZUMA-2, distributions of CD4+ and CD8+ T-cell populations, and frequencies of central effector and memory cell populations after CAR T-cell infusion, were similar among patients previously exposed to ibrutinib or acalabrutinib; however, a trend toward enrichment of Th1/Th17 subpopulations within the CAR+ CD4+ T-cell population and increased prevalence of the Th1 phenotype in peripheral blood mononuclear cells was observed in the ibrutinib cohort [61]. In vitro stimulation of brexu-cel CAR T-cell infusion products with tumor cells led to significant Th1 enrichment in patients exposed to ibrutinib (*P*=0.0058 vs. acalabrutinib) [61], whereas acalabrutinib induced higher levels of Th2 cytokines (e.g., IL-4, IL-5, and IL-13) and granulocyte–macrophage colony-stimulating factor [61]. Preliminary results in patients with R/R MCL from the phase 1 TRANSCEND NHL 001 trial receiving liso-cel showed high response rates at dose levels (DLs) of 50×10^6^ (DL1) and 100×10^6^ (DL2); across both doses, the ORR was 84% with a CR rate of 59% (Table 1) [52]. DL2 was selected for dose expansion [52].

In a US study of real-world outcomes with brexu-cel in 95 patients with R/R MCL, 82% of patients received prior BTKis and 44% were refractory to their last line of anti-lymphoma therapy [53]. With a median follow-up of 6.7 months, the ORR among evaluable patients (*n*=95) was 89%, with 81% achieving CR [53] (Table 1). Among patients with blastoid or pleomorphic MCL, ORR and CR rates were 95% and 87%, respectively. In patients with *TP53* mutations or deletions, rates of ORR and CR were 87% and 71%, respectively; corresponding ORR and CR rates in patients with wild-type *TP53* were 87% and 85%, respectively [53]. Rates of ORR and CR among BTKi–exposed patients were 88% and 79%, respectively; corresponding response rates in the BTKi–naïve subgroup were 94% and 88%, respectively. Rates of 6-month PFS and OS were 66% and 81%, respectively [50••]. In another US multicenter retrospective analysis of 66 patients with R/R MCL who were previously treated with a BTKi and then received brexu-cel after progression on BTKi therapy, the ORR in response-evaluable patients (*n*=56) was 86%, with 77% achieving CR (median follow-up of 4.1 months); rates of 6-month PFS and OS were 77% and 88%, respectively [54]. Similar results were observed in a French registry-based analysis of real-world outcomes in 47 patients with R/R MCL receiving brexu-cel after prior chemoimmunotherapy and a BTKi [56]. At a median follow-up of 3.3 months, ORR among response-evaluable patients was 88%, with 62% achieving CR. Median PFS was 5.3 months and the 6-month PFS rate was 58% [56]. Overall, these studies demonstrate an excellent clinical benefit of CAR T-cell therapy in patients with R/R MCL previously treated with a BTKi and those with high-risk aggressive disease.

### Toxicities Associated with CAR T-Cell Therapy

Frequently observed immune-related AEs associated with CAR T-cell therapy include CRS, neurotoxicities, cytopenias, and infections [62, 63]. Increased cytokine levels due to lymphocyte activation induce symptoms characteristic of CRS (e.g., fever, hypotension, hypoxia, organ dysfunction) [64]. In ZUMA-2, 91% of patients with R/R MCL experienced CRS (grade ≥3, 15%) after CAR T-cell infusion [12••, 59] (Table 1). Neurotoxicity stems from immune effector cell activity and includes toxic encephalopathy with confusion, aplasia, ataxia, delirium, seizures, and cerebral edema [62, 64]. In ZUMA-2, neurotoxicity occurred in 63% of patients (grade ≥3, 31%) [12••]. Grade ≥3 CRS and neurotoxicity were associated with increased T-cell expansion and increased levels of serum granulocyte–macrophage colony-stimulating factor and IL-6; increased IL-2 and IFN-γ levels were also observed with grade ≥3 neurotoxicity [12••]. In ZUMA-2, CRS and neurotoxicity were managed with tocilizumab or glucocorticoids; however, some cases required vasopressor therapy [12••]. In real-world studies of brexu-cel, incidences of CRS and neurotoxicity were generally similar to those observed in the ZUMA-2 trial (Table 1) [50••, 54, 56] and were primarily managed with tocilizumab and corticosteroids [50••, 55]; ICU admission was required in 21–28% of patients [50••, 56]. Recent evidence suggests that early administration of the interleukin 1 receptor antagonist anakinra may reduce the occurrence and severity of CRS and neurotoxicity in patients receiving CAR T-cell therapy [65–67].

Cytopenias generally occur within the first 4 weeks after CAR T-cell infusion and may last up to 3 months [63]. Prolonged cytopenias may be related to CRS severity, tumor burden, prior therapy, and prior hematopoietic stem cell transplantation [49]. Cytopenias were reported in 94% of patients in ZUMA-2; grade ≥3 cytopenias included neutropenia (85%), thrombocytopenia (51%), and anemia (50%) (Table 1) [12••]. Lower rates of grade ≥3 cytopenia (neutropenia, 37%; thrombocytopenia, 43%) were observed in a real-world study of brexu-cel [54] (Table 1).

Patients who receive CAR T-cell therapy are also at risk for infections, with severe CRS being a predisposing risk factor [63]. Bacterial and viral infections occur most frequently during the first few months following CAR T-cell infusion; respiratory tract infections are generally observed after the first 3 months post infusion [63]. Antibiotic or antiviral therapy may be administered prophylactically in patients receiving CAR T-cell therapy [63]. Late-occurring respiratory infections are generally treated on an outpatient basis [63]. In the ZUMA-2 trial, infections occurred in 56% of patients with R/R MCL after administration of brexu-cel; grade ≥3 infections were observed in 32%, with pneumonia (9%) and sepsis (6%) being the most common [12••]. A real-world study of brexu-cel in patients with R/R MCL reported infections in 18% of patients [54].

### Mechanisms of CAR T-Cell Therapy Failure

Failure to respond or loss of response to CAR - cell therapy may occur due to limited T-cell expansion, T-cell exhaustion, T-cell senescence, or surface antigen loss (i.e., CD19 escape) [15, 68]. Use of murine-derived single-chain fragment variable regions in the CAR construct may hinder CAR T-cell expansion in vivo, which in turn increases the likelihood of relapse [15]. Repeated antigen exposure diminishes effector T-cell function leading to T-cell exhaustion [15]. The intracellular costimulatory domains within CAR T-cells allow them to be consumed even in the absence of repeated antigen exposure, and a high tumor burden can accelerate T-cell exhaustion [15]. T-cell senescence stems from continuous activation of CAR T-cells, which results in diminished T-cell effector function with concomitantly high expression of receptors that inhibit T-cell activity (e.g., programmed death 1, cytotoxic T lymphocyte–associated antigen 4, etc.) [15]. Additional features of T-cell senescence include high expression of CD57 or killer cell lectin-like receptor subfamily G1 ligand, which cause CAR T-cells to lose their co-stimulatory signals (e.g., CD28), resulting in the loss of telomerase activity [15]. CD19 escape occurs when patients who relapse after an initial response to CAR T-cell therapy display a similar disease profile that lacks CD19 expression [68]. CD19-negative relapse likely stems from pre-existing CD19-negative clones, lineage conversion, and RNA splicing mechanisms that result in a decrease or loss of CD19 expression [15].

### Rationale for Combined Treatment Strategies with BTKi and CAR T-Cell Therapy

**Fig. 1:**
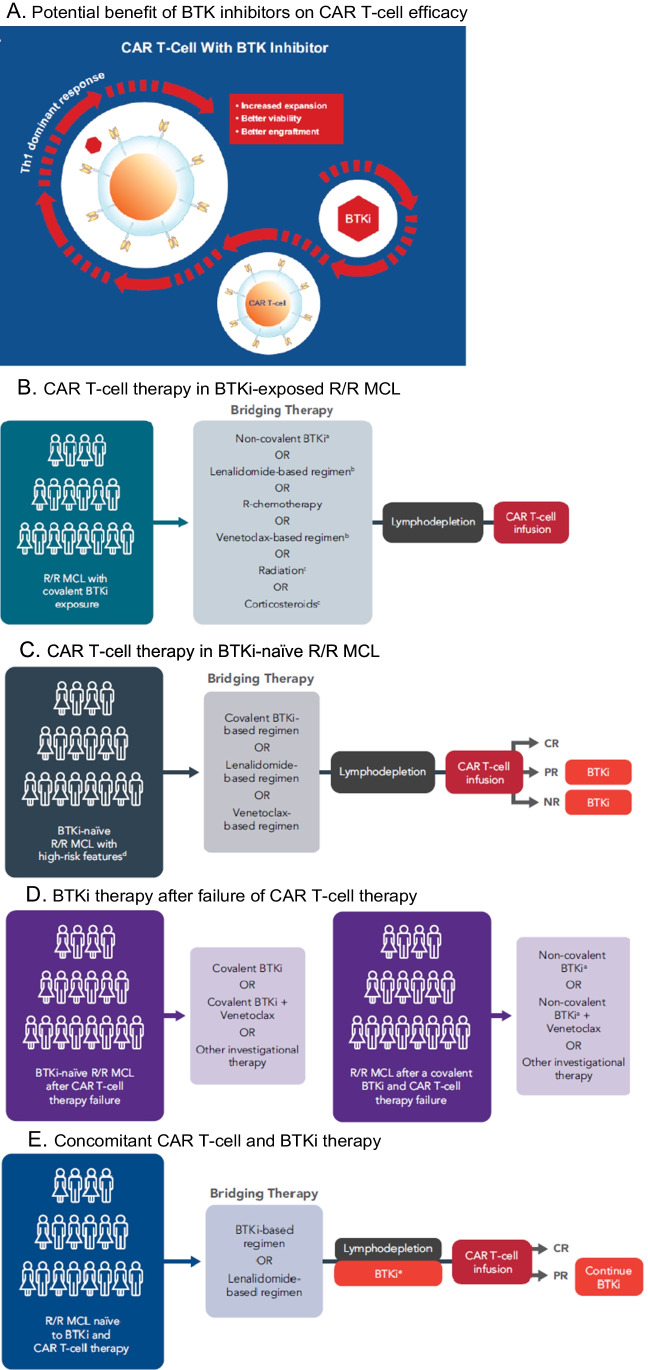
Implementation of BTK inhibitors in CAR T-cell therapy strategies for R/R MCL. (**A**) Potential benefit of BTK inhibitors on CAR T-cell efficacy. (**B**) CAR T-cell therapy in BTKi-exposed R/R MCL. (**C**) CAR T-cell therapy in BTKi-naïve R/R MCL. (**D**) BTKi therapy after failure of CAR T-cell therapy. (**E**) Concomitant CAR T-cell and BTKi therapy.^ a^Non-covalent BTKis have not yet been approved for treatment of MCL.^ b^Bridging therapies in ZUMA-2 were restricted to ibrutinib, acalabrutinib, or corticosteroids; lenalidomide or venetoclax were not permitted but were implemented in a US-based real-world study of brexu-cel in patients with R/R MCL [53]. ^c^Corticosteroids or radiation may be administered alone or in combination with chemoimmunotherapy, radiation, BTKi, or other listed bridging therapy options. ^d^High-risk features include blastoid/pleomorphic phenotype, CNS involvement; *TP53* mutations, or high Ki67 index. ^e^Use of BTK inhibitors in the peri-infusion period has not been established. BTKi, Bruton’s tyrosine kinase inhibitor; CAR, chimeric antigen receptor; CNS, central nervous system; CR, complete response; CRS, cytokine release syndrome; MCL, mantle cell lymphoma; NR, no response; PR, partial response; R, rituximab; R/R, relapsed or refractory

The development of drug resistance or mechanisms that hinder in vivo T-cell expansion or function may diminish the long-term efficacy of BTKis and CAR T-cells, respectively, when administered as monotherapy in B-NHL. There is evidence to suggest BTKi and CAR T-cells may function additively or synergistically when used sequentially or concomitantly (Fig. 1A), although the optimal timing of BTKi administration in the CAR T-cell therapy setting has yet to be established [20]. Harnessing the Th1-dominant effect and ability of a BTKi to penetrate the blood–brain barrier, incorporation of BTKi therapy in patients receiving CAR T-cell therapy could potentiate treatment response. Evidence from studies in CLL suggests that exposure to a BTKi around the time of CAR T-cell infusion may enhance CAR T-cell expansion, viability, and engraftment [16, 17], as well as improve CAR T-cell activation and effector function [21].

Although it is unknown whether sequential administration of BTKi and CAR T-cells yield better outcomes compared with concomitant therapy, results from ZUMA-2, real-world studies, and studies in CLL suggest a benefit with both approaches. Patients with R/R MCL enrolled in ZUMA-2 and real-world studies of brexu-cel demonstrated high response rates after exposure to a BTKi (Table 1) [12••]. Similarly, in a small study of 24 patients with CLL previously treated with ibrutinib (19 had experienced disease progression while receiving ibrutinib and three were intolerant to ibrutinib) [57], administration of CAR T-cell therapy resulted in CR and partial response (PR) rates of 21% and 53%, respectively [57] (Table 1). Administration of liso-cel in the phase 1 dose-escalation part of the TRANSCEND CLL 004 study in patients with R/R CLL or small lymphocytic lymphoma after prior ibrutinib resulted in an ORR of 82% with 45% achieving CR or CR with incomplete bone marrow recovery (Table 1) [58].

Evidence supporting the use of concomitant BTKi and CAR T-cell therapy is mostly limited to studies in CLL, as patients with CLL typically have low rates of CR with CAR T-cell therapy [16], likely due to CLL-induced T-cell dysfunction [69]. In vitro studies suggest that ibrutinib may enhance CAR T-cell expansion and increase cell viability [16, 17] as well as improve cell engraftment, tumor clearance, and survival [17]. Stimulation of CAR T-cells with ibrutinib or acalabrutinib enhanced CAR T-cell effector function; prolonged BTKi stimulation further increased cytokine production and Th1 differentiation. Serial stimulation of CAR T-cells with ibrutinib also enhanced their cytolytic activity [21]. Short-term (48 hours) stimulation of CAR T-cells with ibrutinib reduced IFN-γ production in a dose-dependent manner; acalabrutinib had a variable effect on cytokine production, whereas long-term (6 days) stimulation of CAR T-cells with ibrutinib or acalabrutinib substantially increased IFN-γ production. Stimulation of CAR T-cells with ibrutinib for 18 days increased Th1 differentiation [21].

Fan et al. observed that CAR T-cells derived from patients with CLL were less proliferative than those derived from healthy donors, and that stimulation with ibrutinib significantly increased the expansion and viability of CD3+ T-cells from healthy donors and CLL patients [16]. Ibrutinib-enriched CAR T-cells appeared less differentiated, with a naïve profile and reduced expression of biomarkers indicative of T-cell exhaustion [16]. Similarly, Fraietta and colleagues observed that in patients with CLL, diminished T-cell proliferation was reversed after 5 to 11 cycles of ibrutinib therapy, which was accompanied by improvement in T-cell activation and production of IFN-γ [17]. Patients who achieved CR after 5 to 11 cycles of ibrutinib prior to CAR T-cell infusion had pronounced expansion and engraftment of CTL019 CAR T-cells. In mouse antitumor models, concomitant administration of ibrutinib and CT019 CAR T-cells resulted in increased CAR T-cell engraftment and antitumor activity [17].

In another study, patients with R/R CLL previously exposed to ibrutinib received ibrutinib at least 2 weeks before leukapheresis and continued treatment until up to at least 3 months after CAR T-cell infusion [19]. Concomitant administration of CAR T-cell therapy and ibrutinib resulted in a higher ORR (83%) compared with CAR T-cell therapy alone (56%) (Table 1) [19]. Although CAR T-cell expansion was similar in both groups, patients receiving CAR T-cell therapy with ibrutinib had less severe CRS despite having higher CD4+ T-cell counts [19]. Lower concentrations of monocyte chemoattractant protein 1 and IL-2 receptor alpha were observed in patients who received concomitant ibrutinib and CAR T-cell therapy than in patients who received CAR T-cell therapy alone; IL-6 levels did not differ significantly between the two groups [19]. In a small study of patients with refractory MCL (*n*=3) or refractory follicular lymphoma (*n*=4), ibrutinib in combination with re-administration of CAR T-cell therapy resulted in a CR rate of 86% (*n*=6/7) [18] (Table 1).

Preclinical evaluation of the combined effect of BTKi and CAR T-cell administration in MCL demonstrates similar results to those observed in CLL. Evidence from a study in an MCL cell line (JeKo-1) demonstrates that the investigational noncovalent BTKi vecabrutinib, significantly enhanced CAR T-cell cytotoxicity against MCL CD19+ tumor cells [70]. In the presence of vecabrutnib, IL-6, IL-10, and macrophage inflammatory protein 1β levels were significantly reduced [70]. In mouse JeKo-1 xenograft tumor models, co-administration of CAR T-cells and vecabrutinib resulted in antitumor effects and enhanced CAR T-cell proliferation [70]. RNA sequencing analyses of activated CD19-targeted CAR T-cells showed upregulation of multiple genes associated with PI3K/AKT and Th1 pathways [70]. To date, there are no published clinical data of combination therapy in MCL. Two ongoing clinical trials are investigating the potential benefit of concomitant BTKi and CAR T-cell therapy in patients with R/R MCL. The phase 2 TARMAC trial (NCT04234061) evaluates tisagenlecleucel plus ibrutinib in patients with R/R MCL who failed to achieve CR with ibrutinib or a BTKi–containing regimen for at least 6 months or failed to achieve a PR with a BTKi [71]. Another phase 2 trial is evaluating acalabrutinib in combination with CAR T-cell therapy in patients with R/R MCL currently receiving acalabrutinib with 3 to 7 months of exposure to acalabrutinib prior to screening (NCT04484012) [72].

## Expert Opinion

### Sequential Administration of a BTK Inhibitor and CAR T-Cells

Patients with R/R MCL after exposure to a covalent BTKi should be considered for CAR T-cell therapy (Fig. 1B). Aside from chemoimmunotherapy, other options for bridging therapy include lenalidomide or venetoclax-based regimens [73, 74], radiation, or corticosteroids. In patients who previously received a covalent BTKi, treatment with a non-covalent BTKi may be considered if approved. In patients with BTKi-naïve R/R MCL who have high-risk disease characteristics (e.g., blastoid/pleomorphic phenotype, complex karyotype, *TP53* mutations, high Ki67 index, CNS involvement), CAR T-cell therapy may be administered before BTKi therapy (Fig. 1C), given that these patients are unlikely to have a durable response to a BTKi. Here, a BTKi may be used in bridging therapy regimens and can be reinitiated after CAR T-cell infusion in patients who do not respond or have a partial response to CAR T-cell therapy. Monitoring and management of CRS and neurotoxicity according to institutional protocols is advised.

Patients who relapse or are refractory to CAR T-cell therapy without prior exposure to BTKis may be eligible for treatment with a covalent BTKi alone or in combination with another agent (Fig. 1D) such as venetoclax, which is supported by findings from the safety run-in cohort of the phase 3 SYMPATICO trial [75], or another investigational agent. In patients with prior exposure to a covalent BTKi who relapsed after CAR T-cell therapy (Fig. 1D), treatment with a non-covalent BTKi or another investigational agent may be considered.

### Concomitant Administration of CAR T-Cells and BTK Inhibitors

Because the concomitant use of CAR T-cell therapy and a BTKi has not been approved, standardized guidelines for this approach have not been established. Concomitant CAR T-cell and BTKi therapy may be considered for patients with R/R MCL who are naïve to both CAR T-cell and BTKi therapy (Fig. 1E) because combination therapy may increase treatment efficacy. Based on limited data in patients with CLL, BTKi therapy may be initiated as bridging therapy and continued during lymphodepletion prior to CAR T-cell infusion. Administration of a BTKi during lymphodepletion and immediately after CAR T-cell infusion may result in drug-drug interactions, off-target toxicity and immunodulatory effects, and effects on specific T-cell subsets. In patients receiving ibrutinib, cytokine production could potentially enhance CAR T-cell toxicity and the risk for CRS and immune-mediated neurologic events. Ibrutinib may abrogate the growth of memory T-cells [76], which may affect the efficacy and persistence of CAR T-cell [77]. Although no published evidence to date demonstrates increased toxicity or diminished efficacy with concomitant BTKi therapy during lymphodepletion or immediately after CAR T-cell infusion, patients should be closely monitored for BTKi-related toxicities and other AEs stemming from drug-drug interactions. The cost of adding a BTKi during lymphodepletion should also be considered. If a patient achieves CR without measurable residual disease after CAR T-cell infusion, discontinuation of BTKi treatment may be considered because the benefit of continued BTKi therapy in this setting is unclear; in patients who achieve PR, continued BTKi therapy should be considered.

## Conclusions

Treatment options for R/R MCL have expanded in the past decade with the availability of highly effective BTK inhibitors and the advent of CAR T-cell therapy. Preliminary data supporting the clinical benefit of BTKi/CAR T-cell combinations in R/R CLL warrants similar investigation of BTKi/CAR T-cell combinations in R/R MCL. Ongoing trials (NCT04234061 and NCT04484012) evaluating concomitant BTKi and CAR T-cell therapy approaches in R/R MCL will help determine the potential benefit of the BTKi/CAR T-cell combination in R/R MCL. Furthermore, BTKi/CAR T-cell combinations for MCL should be explored in the frontline setting for high-risk disease. As treatment of R/R MCL continues to rapidly evolve with emerging therapies against ROR1 and other key targets, combination treatment strategies with established and novel agents will improve outcomes in R/R MCL.

## References

[1] Jain P, Wang ML. Mantle cell lymphoma—a comprehensive update on molecular pathogenesis, risk stratification, clinical approach, and current and novel treatments. Am J Hematol. 2022;97(5):638–56. 10.1002/ajh.26523 35266562

[2] Wen T, Wang J, Shi Y, Qian H, Liu P (2021). Inhibitors targeting Bruton's tyrosine kinase in cancers: drug development advances. Leukemia..

[3] Wang ML, Blum KA, Martin P, Goy A, Auer R, Kahl BS (2015). Long-term follow-up of MCL patients treated with single-agent ibrutinib: updated safety and efficacy results. Blood..

[4] Rule S, Jurczak W, Jerkeman M, Rusconi C, Trneny M, Offner F (2018). Ibrutinib versus temsirolimus: 3-year follow-up of patients with previously treated mantle cell lymphoma from the phase 3, international, randomized, open-label RAY study. Leukemia..

[5] Gu D, Tang H, Wu J, Li J, Miao Y (2021). Targeting Bruton tyrosine kinase using non-covalent inhibitors in B cell malignancies. J Hematol Oncol.

[6] Jaglowski SM, Blazar BR (2018). How ibrutinib, a B-cell malignancy drug, became an FDA-approved second-line therapy for steroid-resistant chronic GVHD. Blood Adv.

[7] Dubovsky JA, Beckwith KA, Natarajan G, Woyach JA, Jaglowski S, Zhong Y (2013). Ibrutinib is an irreversible molecular inhibitor of ITK driving a Th1-selective pressure in T lymphocytes. Blood..

[8] Wu J, Zhang M, Liu D (2016). Acalabrutinib (ACP-196): a selective second-generation BTK inhibitor. J Hematol Oncol.

[9] Guo Y, Liu Y, Hu N, Yu D, Zhou C, Shi G (2019). Discovery of zanubrutinib (BGB-3111), a novel, potent, and selective covalent inhibitor of Bruton's tyrosine kinase. J Med Chem.

[10] Wang M, Rule S, Zinzani PL, Goy A, Casasnovas O, Smith SD (2018). Acalabrutinib in relapsed or refractory mantle cell lymphoma (ACE-LY-004): a single-arm, multicentre, phase 2 trial. Lancet..

[11] Song Y, Zhou K, Zou D, Zhou J, Hu J, Yang H, et al. Zanubrutinib in relapsed/refractory mantle cell lymphoma: long-term efficacy and safety results from a phase 2 study. Blood. 2022. 10.1182/blood.2021014162. 10.1182/blood.2021014162 PMC9136878 35303070

[12] Wang M, Munoz J, Goy A, Locke FL, Jacobson CA, Hill BT (2020). KTE-X19 CAR T-cell therapy in relapsed or refractory mantle-cell lymphoma. N Engl J Med.

[13] Jain P, Kanagal-Shamanna R, Zhang S, Ahmed M, Ghorab A, Zhang L (2018). Long-term outcomes and mutation profiling of patients with mantle cell lymphoma (MCL) who discontinued ibrutinib. Br J Haematol.

[14] Ondrisova L, Mraz M (2020). Genetic and non-genetic mechanisms of resistance to BCR signaling inhibitors in B cell malignancies. Front Oncol.

[15] Li X, Chen W (2019). Mechanisms of failure of chimeric antigen receptor T-cell therapy. Curr Opin Hematol.

[16] Fan F, Yoo HJ, Stock S, Wang L, Liu Y, Schubert ML (2021). Ibrutinib for improved chimeric antigen receptor T-cell production for chronic lymphocytic leukemia patients. Int J Cancer.

[17] Fraietta JA, Beckwith KA, Patel PR, Ruella M, Zheng Z, Barrett DM (2016). Ibrutinib enhances chimeric antigen receptor T-cell engraftment and efficacy in leukemia. Blood..

[18] Liu M, Deng H, Mu J, Li Q, Pu Y, Jiang Y (2021). Ibrutinib improves the efficacy of anti-CD19-CAR T-cell therapy in patients with refractory non-Hodgkin lymphoma. Cancer Sci.

[19] Gauthier J, Hirayama AV, Purushe J, Hay KA, Lymp J, Li DH (2020). Feasibility and efficacy of CD19-targeted CAR T-cells with concurrent ibrutinib for CLL after ibrutinib failure. Blood..

[20] Jacobson CA, Maus MV (2020). C(h)AR-ting a new course in incurable lymphomas: CAR T-cells for mantle cell and follicular lymphomas. Blood Adv.

[21] Qin JS, Johnstone TG, Baturevych A, Hause RJ, Ragan SP, Clouser CR (2020). Antitumor potency of an anti-CD19 chimeric antigen receptor T-cell therapy, lisocabtagene maraleucel in combination with ibrutinib or acalabrutinib. J Immunother.

[22] Hanel W, Epperla N (2020). Emerging therapies in mantle cell lymphoma. J Hematol Oncol.

[23] Dreyling M, Jurczak W, Jerkeman M, Silva RS, Rusconi C, Trneny M (2016). Ibrutinib versus temsirolimus in patients with relapsed or refractory mantle-cell lymphoma: an international, randomised, open-label, phase 3 study. Lancet..

[24] Wang M, Rule S, PL ZI, Goy AH, Casasnovas R-O, Smith SD (2020). Acalabrutinib monotherapy in patients with relapsed/refractory mantle cell lymphoma: long-term efficacy and safety results from a phase 2 study. Blood..

[25] Mato AR, Shah NN, Jurczak W, Cheah CY, Pagel JM, Woyach JA (2021). Pirtobrutinib in relapsed or refractory B-cell malignancies (BRUIN): a phase 1/2 study. Lancet..

[26] Wang ML, Shah N, Alencar A, Gerson JN, Patel MR, Fakhri B, et al. Pirtobrutinib, a highly selective, non-covalent, (reversible) BTK inhibitor in previously treated mantle cell lymphoma: updated results from the phase 1/2 BRUIN study. Blood. 2021;138(Suppl 1):381.

[27] Mannina D, Loteta B (2017). Ibrutinib treatment of mantle cell lymphoma relapsing at central nervous system: a case report and literature review. Case Rep Hematol.

[28] Bernard S, Goldwirt L, Amorim S, Brice P, Briere J, de Kerviler E (2015). Activity of ibrutinib in mantle cell lymphoma patients with central nervous system relapse. Blood..

[29] Zhang Y, Li Y, Zhuang Z, Wang W, Wei C, Zhao D (2021). Preliminary evaluation of zanubrutinib-containing regimens in DLBCL and the cerebrospinal fluid distribution of zanubrutinib: a 13-case series. Front Oncol.

[30] Estupiňan HY, Berglof A, Zain R, Smith CIE (2021). Comparative analysis of BTK inhibitors and mechanisms underlying adverse effects. Front Cell Dev Biol.

[31] Abbas HA, Wierda WG (2021). Acalabrutinib: a selective Bruton tyrosine kinase inhibitor for the treatment of B-cell malignancies. Front Oncol.

[32] Trotman J, Opat S, Gottlieb D, Simpson D, Marlton P, Cull G (2020). Zanubrutinib for the treatment of patients with Waldenström macroglobulinemia: 3 years of follow-up. Blood..

[33] Shatzel JJ, Olson SR, Tao DL, McCarty OJT, Danilov AV, DeLoughery TG (2017). Ibrutinib-associated bleeding: pathogenesis, management and risk reduction strategies. J Thromb Haemost.

[34] Kamel S, Horton L, Ysebaert L, Levade M, Burbury K, Tan S (2015). Ibrutinib inhibits collagen-mediated but not ADP-mediated platelet aggregation. Leukemia..

[35] Abdel-Qadir H, Sabrie N, Leong D, Pang A, Austin PC, Prica A (2021). Cardiovascular risk associated with ibrutinib use in chronic lymphocytic leukemia: a population-based cohort study. J Clin Oncol.

[36] Salem JE, Manouchehri A, Bretagne M, Lebrun-Vignes B, Groarke JD, Johnson DB (2019). Cardiovascular toxicities associated with ibrutinib. J Am Coll Cardiol.

[37] Rule S, Dreyling M, Goy A, Hess G, Auer R, Kahl B (2017). Outcomes in 370 patients with mantle cell lymphoma treated with ibrutinib: a pooled analysis from three open-label studies. Br J Haematol.

[38] McFarlane T, Rehman N, Wang K, Lee J, Carter C (2020). Cutaneous toxicities of new targeted cancer therapies: must know for diagnosis, management, and patient-proxy empowerment. Ann Palliat Med.

[39] Tam CS, Opat S, D'Sa S, Jurczak W, Lee HP, Cull G (2020). A randomized phase 3 trial of zanubrutinib vs ibrutinib in symptomatic Waldenström macroglobulinemia: the ASPEN study. Blood..

[40] Lionakis MS, Dunleavy K, Roschewski M, Widemann BC, Butman JA, Schmitz R (2017). Inhibition of B cell receptor signaling by ibrutinib in primary CNS lymphoma. Cancer Cell.

[41] Ghez D, Calleja A, Protin C, Baron M, Ledoux MP, Damaj G (2018). Early-onset invasive aspergillosis and other fungal infections in patients treated with ibrutinib. Blood..

[42] Handunetti SM, Sang Tang CP, Nguyen T, Zhou X, Thompson E, Sun H (2019). BTK Leu528Trp - a potential secondary resistance mechanism specific for patients with chronic lymphocytic leukemia treated with the next generation BTK inhibitor zanubrutinib. Blood..

[43] Woyach JA, Huang Y, Rogers K, Bhat SA, Grever MR, Lozanski A (2019). Resistance to acalabrutinib in CLL is mediated primarily by BTK mutations. Blood..

[44] Zhang S, Jiang VC, Han G, Hao D, Lian J, Liu Y (2021). Longitudinal single-cell profiling reveals molecular heterogeneity and tumor-immune evolution in refractory mantle cell lymphoma. Nat Commun.

[45] Smith CIE, Burger JA (2021). Resistance mutations to BTK inhibitors originate from the NF-kappaB but not from the PI3K-RAS-MAPK arm of the B cell receptor signaling pathway. Front Immunol.

[46] Rauert-Wunderlich H, Rudelius M, Berberich I, Rosenwald A (2018). CD40L mediated alternative NFkappaB-signaling induces resistance to BCR-inhibitors in patients with mantle cell lymphoma. Cell Death Dis.

[47] Feins S, Kong W, Williams EF, Milone MC, Fraietta JA (2019). An introduction to chimeric antigen receptor (CAR) T-cell immunotherapy for human cancer. Am J Hematol.

[48] Vitale C, Strati P (2020). CAR T-cell therapy for B-cell non-Hodgkin lymphoma and chronic lymphocytic leukemia: clinical trials and real-world experiences. Front Oncol.

[49] Jain T, Bar M, Kansagra AJ, Chong EA, Hashmi SK, Neelapu SS (2019). Use of chimeric antigen receptor T-cell therapy in clinical practice for relapsed/refractory aggressive B cell non-hodgkin lymphoma: an expert panel opinion from the American Society for Transplantation and Cellular Therapy. Biol Blood Marrow Transplant.

[50] •• Wang Y, Jain P, Locke FL, Munoz J, Maurer MJ, Beitinjaneh A, et al. Brexucabtagene autoleucel for relapsed/refractory mantle cell lymphoma: real world experience from the US Lymphoma CAR T Consortium. Blood. 2021;138. **This real-world study showed that brexucabtagene autoleucel can be prescribed as standard of care with similar efficacy and toxicity as compared with the pivotal clinical trial (ZUMA-2) experience.**

[51] Wang M, Munoz J, Goy AH, Locke FL, Jacobson CA, Hill BT (2020). One-year follow-up of ZUMA-2, the multicenter, registrational study of KTE-X19 in patients with relapsed/refractory mantle cell lymphoma. Blood..

[52] Palomba ML, Gordon LI, Siddiqi T, Abramson JS, Kamdar M, Lunning MA (2020). Safety and preliminary efficacy in patients with relapsed/refractory mantle cell lymphoma receiving lisocabtagene maraleucel in Transcend NHL 001. Blood..

[53] Wang Y, Jain P, Locke FL, Munoz J, Maurer MJ, Beitinjaneh A, et al. Brexucabtagene autoleucel for relapsed/refractory mantle cell lymphoma: real world experience from the US Lymphoma CAR T Consortium. Blood. 2021;138(Suppl 1):744.

[54] Romancik JT, Goyal S, Gerson JN, Ballard HJ, Sawalha Y, Bond DA, et al. Analysis of outcomes and predictors of response in patients with relapsed mantle cell lymphoma treated with brexucabtagene autoleucel. Blood. 2021;138(Suppl 1):1756.

[55] Romancik JT, Goyal S, Gerson JN, Ballard HJ, Sawalha Y, Bond DA (2021). Analysis of outcomes and predictors of response in patients with relapsed mantle cell lymphoma treated with brexucabtagene autoleucel. Blood..

[56] Herbaux G, Bret C, Di Blasi R, Bachy E, Beauvais D, Gat E (2021). Kte-X19 in relapsed or refractory mantle-cell lymphoma, a "real-life" study from the Descar-T Registry and Lysa Group. Blood..

[57] Turtle CJ, Hay KA, Hanafi LA, Li D, Cherian S, Chen X (2017). Durable molecular remissions in chronic lymphocytic leukemia treated with CD19-specific chimeric antigen receptor-modified T-cells after failure of ibrutinib. J Clin Oncol.

[58] Siddiqi T, Soumerai JD, Dorritie KA, Stephens DM, Riedell PA, Arnason JE, et al. Phase 1 TRANSCEND CLL 004 study of lisocabtagene maraleucel in patients with relapsed/refractory CLL or SLL. Blood. 2022;139(12):1794–1806.10.1182/blood.202101189534699592

[59] ClinicalTrials.gov. Study to evaluate the efficacy of brexucabtagene autoleucel (KTE-X19) in participants with relapsed/refractory mantle cell lymphoma (ZUMA-2). https://clinicaltrials.gov/ct2/show/NCT02601313. Accessed November 3, 2021.

[60] Wang M, Rossi JM, Munoz J, Goy AH, Locke FL, Reagan PM (2020). Pharmacological profile and clinical outcomes of KTE-X19 by prior Bruton tyrosine kinase inhibitor (BTKi) exposure or mantle cell lymphoma (MCL) morphology in patients with relapsed/refractory (R/R) MCL in the ZUMA-2 trial. Blood.

[61] Scarfo I, Gallagher KME, Leick MB, Kann NC, Budka J, Sowirajan B (2021). Effects of prior exposure to Tec kinase (BTK/ITK) inhibitors on Kte-X19 products. Blood..

[62] Lee DW, Santomasso BD, Locke FL, Ghobadi A, Turtle CJ, Brudno JN (2019). ASTCT consensus grading for cytokine release syndrome and neurologic toxicity associated with immune effector cells. Biol Blood Marrow Transplant.

[63] Yanez L, Alarcon A, Sanchez-Escamilla M, Perales MA (2020). How I treat adverse effects of CAR-T-cell therapy. ESMO Open.

[64] Rafiq S, Hackett CS, Brentjens RJ (2020). Engineering strategies to overcome the current roadblocks in CAR T-cell therapy. Nat Rev Clin Oncol.

[65] Park JH, Sauter CS, Palomba ML, Shah GL, Dahi PB, Lin RJ (2021). A phase II study of prophylactic anakinra to prevent CRS and neurotoxicity in patients receiving CD19 CAR T-cell therapy for relapsed or refractory lymphoma. Blood..

[66] Wong SW, Richard S, Lin Y, Madduri D, Jackson CC, Zudaire E (2021). Anakinra targeting cytokine release syndrome associated with chimeric antigen receptor T-cell therapies. Blood..

[67] Frigault MJ, Gallagher KME, Wehrli M, Valles B, Casey K, Lindell K, et al. A phase II trial of anakinra for prevention of CAR-T-cell mediated neurotoxicity. Blood. 2021;138(Suppl 1):2814.

[68] Majzner RG, Mackall CL (2018). Tumor antigen escape from CAR T-cell therapy. Cancer Discov.

[69] Peters FS, Strefford JC, Eldering E, Kater AP (2021). T-cell dysfunction in chronic lymphocytic leukemia from an epigenetic perspective. Haematologica..

[70] Adada MM, Sakemura R, Cox MJ, Manriquez-Roman C, Siegler EL, Tapper E (2021). Favorable modulation of chimeric antigen receptor T-cells safety and efficacy by the non-covalent BTK inhibitor vecabrutinib. Blood..

[71] ClinicalTrials.gov. Clinical Trial to Assess The Efficacy and Safety of the Combination of Tisagenlecleucel And Ibrutinib in Mantle Cell Lymphoma (TARMAC). https://clinicaltrials.gov/ct2/show/NCT04234061. Accessed October 27, 2021.

[72] ClinicalTrials.gov. A Phase 2 Study to Evaluate CD19-Specific Chimeric Antigen Receptor(CAR)-T-Cells Combined With Acalabrutinib for Patients With Relapsed or Refractory Mantle Cell Lymphoma (MCL). https://clinicaltrials.gov/ct2/show/NCT04484012. Accessed December 20, 2021.

[73] Romancik JT, Cohen JB (2021). Sequencing of novel therapies for mantle cell lymphoma. Curr Treat Options in Oncol.

[74] Eyre TA, Walter HS, Iyengar S, Follows G, Cross M, Fox CP (2019). Efficacy of venetoclax monotherapy in patients with relapsed, refractory mantle cell lymphoma after Bruton tyrosine kinase inhibitor therapy. Haematologica..

[75] Wang M, Ramchandren R, Chen R, Karlin L, Chong G, Jurczak W (2021). Concurrent ibrutinib plus venetoclax in relapsed/refractory mantle cell lymphoma: the safety run-in of the phase 3 SYMPATICO study. J Hematol Oncol.

[76] Solman IG, Blum LK, Hoh HY, Kipps TJ, Burger JA, Barrientos JC (2020). Ibrutinib restores immune cell numbers and function in first-line and relapsed/refractory chronic lymphocytic leukemia. Leuk Res.

[77] McLellan AD, Ali Hosseini Rad SM (2019). Chimeric antigen receptor T-cell persistence and memory cell formation. Immunol Cell Biol.

